# Identification of thermo tolerant lactic acid bacteria isolated from silage prepared in the hot and humid climate of Southwestern Japan

**DOI:** 10.1186/2193-1801-2-485

**Published:** 2013-09-25

**Authors:** Katsumi Doi, Yousuke Nishizaki, Hidetoshi Kimura, Maki Kitahara, Yasuhiro Fujino, Sadahiro Ohmomo, Toshihisa Ohshima, Seiya Ogata

**Affiliations:** Microbial Genetic Division, Institute of Genetic Resources, Faculty of Agriculture, Kyushu University, 6-10-1 Hakozaki, Higashi-ku, Fukuoka, 812-8581 Japan; Faculty of Arts and Science, Kyushu University, 744 Motooka, Nishi-ku, Fukuoka, 819-0395 Japan; Microbe Division, Japan Collection of Microorganisms, RIKEN BioResource Center, 3-1-1 Koyadai, Tsukuba, Ibaraki, 305-0074 Japan; Asama chemical Co., Ltd., 20-3 Nihonbashi-Kodenma-cho, Chuo-ku, Tokyo, 103-0001 Japan

**Keywords:** Lactic acid bacteria, *Lactobacillus*, *Pediococcus*, Silage, Hot and humid environment

## Abstract

To develop high-quality silage starters adapted to hot and humid weather, 12 LAB isolates from silage produced in Kyushu and Okinawa, Japan were characterized based on their morphological features, growth curves and sugar utilization. In addition, the nucleotide sequences of the V2-V3 region of their 16S rRNA genes and the 16S-23S rRNA intergenic spacer (ITS) regions were determined. The isolates were also subjected to RAPD-PCR analysis, DNA-DNA hybridization, G+C content analysis and immuno-identification using species-specific monoclonal antibodies and SDS-PAGE profiling. Nearly all of the isolates exhibited high thermotolerance and rapid growth. Combining ITS sequence analysis, RAPD-PCR and immuno-identification enabled rapid and accurate identification of closely related LAB strains that other methods failed to appropriately differentiate; for example, *L. plantarum* was distinguished from *L. pentosus,* and *L. casei* was distinguished from *L. rhamonsus.* Using the aforementioned techniques, the isolated strains were identified as *L. plantarum*, *L. rhamonsus, L. rapi, Pediococcus pentosaceus* and *P. lolii*. Our findings also showed that there is greater diversity among thermophilic LABs in silage prepared in a hot and humid environment.

## Introduction

Silage is a storable fodder typically produced through a sequential microbial process involving homofermentative lactic acid bacteria (LAB) such as *Lactobacillus plantarum, Lactococcus lactis, Leuconostoc pseudomesenteroides, Pediococcus acidilactici* and *Enterococcus faecalis* (Cai et al. 1999; Ennahar et al. 2003; Giraffa et al. 2010). In recent years, the demand for dairy products has increased in many developing countries in the tropical and subtropical regions of Asia and Africa; however, production of the silage necessary for dairy farming has been hindered in these regions because the ensiling process is dependent on local environmental conditions (Namihira et al. 2010). For example, virtually all of the many available silage starters (Holzer et al. 2003; Yan et al. 2008) are hampered by high temperatures and humidity (Ohmomo et al. 2002; Mulrooney and Kung 2008). This is in part because the phage infections that occur in hot humid weather reduce the viability of LAB (Kaneshige et al. 1994). At present, therefore, there is a strong need to identify starter strains suitable for use in hot climates.

The islands of Kyushu and Okinawa in southwestern Japan have a humid, subtropical climate. Production of high-quality silage there is difficult due to unbroken periods of extremely hot days, with temperatures exceeding 30°C in summer, and to the high moisture content (over 80%) in the plant materials. Stable production of high-quality silage in these regions will require the identification and application of acid-tolerant, thermophilic LAB or homolactic acid fermented LAB as starter strains (Mulrooney and Kung 2008). Given the limits of the available technology, however, the screening, selection and construction of starter cultures for silage making remains a challenge, as classification of isolated strains is still difficult. In particular, closely related species, such as *L. plantarum*, *L. casei*, *L. rhanmosus* and *L. pentosus*, which are the main dominant strains in silage, are difficult or impossible to distinguish on the basis of their phenotypes and genotypes (Hammes and Hertel 2006). Some PCR amplification methods for the 16S-23S rRNA, *recA* and *dnaK* genes have been used to identify *L. plantarum*-related species (Torriani et al. 2001; Huang et al. 2010; Ouoba et al. 2010), but there is no generally accepted or systematic approach to identification of silage-making lactobacilli. In particular, there have been few studies characterizing LAB strains grown in silage produced in tropical or subtropical regions (Namihira et al. 2010).

Here, we report the isolation and characterization of LAB from silage produced in Okinawa, Japan. We used various identification methods to assess the diversity of silage-making LAB in subtropical areas, and to identify species closely related to *L. plantarum* and *L. pentosus* and to *L. casei* and *L. rhamonsus.* Our findings represent the first use of immuno-identification for the identification of LAB from silage.

## Materials and methods

### Bacterial strains and culture conditions

Twelve representative LAB strains isolated from silage prepared in Okinawa prefecture, Japan were used. *L. brevis* JCM 1059^T^, *L. casei* JCM 1134^T^, *L. fermentum* JCM 1173^T^, *L. kefiri* JCM 5818^T^, *L. parakefiri* JCM 8573^T^, *L. paraplantarum* JCM 12533^T^*, L. plantarum* JCM 1057, *L. plantarum* JCM 1149^T^, *L. rapi* JCM 15042^T^, *L. rhanmosus* JCM 1136^T^, *L. pentosus* JCM 1558^T^, *Pediococcus acidilactici* JCM 8797^T^ and *P. pentosaceus* JCM 5890^T^ were used as reference strains. *Escherichia coli* DH5α was used as a host strain for plasmid vector pTA2 (Toyobo Co., Ltd., Osaka, Japan).

Bacto™ Lactobacilli MRS Broth (Becton, Dickinson and Company, NJ, USA) and agar plates (2.0% agar) were used for transplantation and growth of *Lactobacillus* strains. GAM Broth (Nissui Pharmaceutical Co., Tokyo, Japan) and R-CW agar (Kojima et al. 1993) were used for growth of *P. pentosaceus* JCM 5890^T^ and *L. parakefir* JCM 8573^T^, respectively. *L. kefir* JCM 5818^T^, *L. parakefir* JCM 8573^T^, *L. pentosus* JCM 1558^T^, *L. plantarum* JCM 1057, *L. plantarum* JCM 1149^T^ and *P. acidilactici* JCM 8797^T^ were cultivated at 30°C under anaerobic conditions. Cultivation of other strains was carried out at 37°C under anaerobic conditions. A microaerobic condition (5% O_2_, 10% CO_2_, 85% N_2_) for cultivation of *L. paraplantarum* ATCC 700211^T^ was generated using AnaeroPack Plus (Mitsubishi Gas Chemical Co., Tokyo, Japan). LB broth and agar plates (2.0%) were used for growth and transformation of *E. coli*. LB plates containing ampicillin (100 μg/ml), isopropyl-β-d-thiogalactopyranoside (120 Dμg/ml) and 5-bromo-4-chloro-3-indolyl-β-d-galactopyranoside (400 Dμg /ml) were used for selection of blue/white colonies.

### Physiological characterization

The shapes of all isolates were observed using an Axioskop phase-contrast microscope (Carl Zeiss, Oberkochen, Germany) and transmission electron microscopy (JEM 2000 EX; JEOL, Tokyo, Japan). Gas formation from glucose, fermentation type, lactic acid production, and peptidoglycan type were examined as described previously (Tanaka et al. 1994). In addition, the characteristics of the carbohydrate fermentation by all strains were determined using an API 50CHL kit (BioMérieux, Montalieu Vercie, France). Growth curves for the isolates were constructed using Miniphoto 518 (TAITEC Corporation, Saitama, Japan) by monitoring the optical density at 660 nm.

### Total DNA extraction

Total DNA was isolated from LAB strains cultured overnight in 600 ml of liquid medium supplemented with 0.5% glycine. After washing the cells with wash buffer (50 mM Tris–HCl, 150 mM NaCl, 100 mM EDTA; pH 8.0), they were collected by centrifugation and lysed in 10 ml of lysis buffer (50 mM Tris–HCl, 10 mM EDTA, 10 mg/ml lysozyme (Seikagaku Corporation, Tokyo, Japan), 2 mg/ml N-acetylmuramidase SG (Seikagaku Corporation), 100 μg/ml RNase A; pH 8.0). The lysate was then added to 200 ml of 25% sodium dodecyl sulfate (SDS) and incubated for 2 h at 37°C, after which the DNA was extracted first in phenol-chloroform-isoamyl alcohol (25:24:1) and then in chloroform-isoamyl alcohol (24:1). The extracted DNA was then precipitated in isopropanol, collected and dissolved in 100 ml of TE buffer.

### PCR amplification and cloning amplified fragments

The primers used in this study are listed in Table 1. To amplify the V2-V3 regions of 16S rRNA and the 16S-23S intergenic spacer (ITS) region, primers were designed as described by Tannock et al. (1999). With all primer sets, PCR was carried out in a 50-μl reaction volume containing 5 U of KOD DNA polymerase (Toyobo), 5 μl of 10×PCR reaction buffer, 0.2 mM each primer, 0.2 mM dNTPs, 0.5 mM MgCl_2_ and 200 ng of purified genomic DNA. The amplified products were separated on 2.0% agarose gels containing 1×TAE electrophoresis buffer, purified using a Cyclo-Pure Gel Extraction Kit (AMRESCO Inc., Solon, OH, USA) according to the manufactures' instructions, and then ligated to pTA2 vector using a Takara BKL Kit (Takara Bio Inc., Shiga, Japan). The recombinant DNAs to be transformed into *E. coli* DH5α competent cells were extracted and purified through alkaline lysis using QIAGEN-tip 20 (QIAGEN GmbH, Hilden, Germany).

**Table 1:** **Primers used in this study**

Primer	Sequence (5’ to 3’)	Use	Specificity or target	Reference or source
HDA1	ACTCCTACGGGAGGCAGCAGT	PCR	V2-V3 region of 16SrRNA	(Tannock *et al.* 1999)
HDA2	GTATTACCGCGGCTGCTGGCAC	PCR	V2-V3 region of 16SrRNA	(Tannock *et al.* 1999)
16A-1	GAATCGCTAGTAATCG	PCR	16S-23S intergeneic spacer region	(Tannock *et al.* 1999)
23B-1	GGGTTCCCCCATTCGGA	PCR	16S-23S intergeneic spacer region	(Tannock *et al.* 1999)
M13 Primer M3	GTAAA ACGACGGCCAGT	DNA sequence	Plasmid vector pTA2	TaKaRa Bio
M13 Primer RV	CAGGA AACAGCTATGAC	DNA Sequence	Plasmid vector pTA2	TaKaRa Bio
AP-A-2	TGGATTGGTC	RAPD		This study
AP-A-6	AAACTCCGTC	RAPD		This study
AP-A-8	TGGTAAAGGG	RAPD		This study
AP-A-19	GATCATAGCC	RAPD		This study
AP-A-23	GATCTGACTG	RAPD		This study

### Nucleotide sequence analysis

Nucleotide sequences were determined using a Thermo Sequenase fluorescently labeled primer cycle sequencing kit with 7-deaza-dGTP (GE Healthcare Bio-Sciences, Uppsala, Sweden) and ALF express II DNA sequencer. The sequences of the amplified DNA were scanned based on data registered in databases using GENETIX-MAC ver. 15 (GENETYX, Tokyo, Japan). Comparisons between the sequences and the databases were made using the Blast program (http://blast.ncbi.nlm.nih.gov/Blast.cgi). Multiple alignments and phylogenetic analyses were accomplished using ClustalW ver. 2.1 (http://clustalw.ddbj.nig.ac.jp/) and the neighbor-joining (NJ) method. Evolutionary distance values were estimated using Kimura’s 2 parameter method.

### RAPD analysis

RAPD-PCR analyses were performed as described previously (Fujino et al. 2008) using a single 10-mer random primer listed in Table 1 and 0.5 U of Gene Taq FP polymerase (Nippongene, Toyama, Japan). The PCR protocol entailed initial denaturation at 95°C for 5 min, followed by 35 cycles of 1 min denaturation at 95°C, annealing 36°C for 1 min and extension for 2 min at 72°C, and a final extension for 5 min at 72°C. RAPD-PCR products were analyzed by electrophoresis run on 2.0% agarose gels in 1X TAE for 1 h at 100 V.

### SDS-PAGE profiling

SDS-PAGE was carried out using an AE-6200 electrophoresis unit (ATTO, Tokyo, Japan) to determine the size and number of whole proteins in the isolates. Electrophoresis was carried using the method of Laemmli (1970). Samples were boiled for 10 min at 100°C in SDS-PAGE buffer (125 mM Tris–HCl (pH 6.8), 10% 2-mercaptoethanol, 4% SDS, 10% glycerol, 0.004% bromophenol blue) to ensure denaturation before being loaded onto the gel. Each well was loaded with 20 μl of sample containing approximately 10 μg of protein. After electrophoresis, the gels were stained with Coomassie blue R-250, destained using 7.5% glacial acetic acid and 5% methanol, and then photographed. Numerical analysis of the protein pattern was performed using the GelCompar system (version 4.0; Applied Maths, Sint-Martens-Latem, Belgium), which normalizes fragment pattern data for band intensity, after which the relative band position is compared to molecular weight standards. The similarities between all pairs were expressed using a pair-group method with arithmetic averages (UPGMA) for construction of the dendrogram. All strains were analyzed at least three times to ensure the reproducibility of the fingerprinting pattern.

### Immuno-identification

For identification using monoclonal antibodies, anti-*L. casei* monoclonal antibody (72-6D2) and anti**-***L. plantarum* monoclonal antibody (55-1C3) (Asahi Food & Healthcare Co., ltd., Tokyo, Japan) were used as primary antibodies, and an affinity purified, horseradish peroxidase-conjugated rabbit anti-mouse IgG_1_ (MP Biomedicals, Solon, OH, USA) was used as the secondary antibody. LAB were incubated until late log growth phase and washed with TES buffer (50 mM NaCl, 30 mM Tris–HCl, 5 mM EDTA; pH 8.0), after which they were suspended in TES buffer at an optical density of 1.0 at 660 nm. Aliquots (100 ml) of the suspension were then incubated overnight at 4°C in a 96-well microtiter plate. To determine the dose response relationship, various numbers of bacteria were inoculated onto similar microtiter plates. After washing with Tween-PBS (phosphate-buffered saline (137 mM NaCl, 2.7 mM KCl, 8.1 mM Na_2_HPO_4_ and 1.5 mM KH_2_PO_4_) supplemented with 0.1% Tween 20), the plates were blocked by incubation for 1 h at 37°C with 120 ml of carbonate buffer containing 1% bovine serum albumin (BSA). The plates were then washed three times and treated for 2 h at 37°C with 100 ml per well of appropriate dilutions of monoclonal antibodies in Tween-PBS with 1% BSA. The plates were then washed again and treated for 2 h at 37°C with 100 ml of a 1:1000 dilution of secondary antibody in Tween-PBS with 1% BSA. After washing, the plates were treated with 100 ml of the coloring agent (100 mM citric acid, 200 mM Na_2_HPO_4_, 10% DMSO containing 2% 3, 3', 5, 5', tetramethylbenzidine, 0.02% H_2_O_2_ (30% aqueous); pH 6.2) for 10 min at 37°C and finally with 50 ml per well of 2 M sulfuric acid to stop the reaction.

### G+C content and DNA-DNA hybridization

To determine the genomic G+C content, extracted DNA was purified by ultracentrifugation (Optima TMX Ultracentrifuge, Beckman Coulter Inc.) for 24 h at 80,000×*g* in cesium chloride (1 g/ml) and ethidium bromide (0.8 mg/ml). The DNA was then dissolved in ultrapure distilled water, and the ethidium bromide was removed with isopropanol, after which the G+C content of the purified DNA was determined using DNA-GC kits (Yamasa-Shouyu, Chiba, Japan) according to the manufacturer’s instructions. The nucleoside mixture obtained was then separated by HPLC (model 600E, Waters Corporation, Massachusetts, MA, USA) using a YMC pack AQ-312 column (YMC Co., Ltd., Kyoto, Japan). The mobile phase consisted of 10 mM H_3_PO_4_ and 10 mM KH_2_PO_4_ (pH3.5). As a control, genomic DNA from phage λ was analyzed along with the sample.

DNA-DNA hybridization was carried out as described previously (Ezaki et al. 1989; Adnan et al. 1993). Samples of purified DNA from each of the 12 isolated and 6 type strains were suspended in TE buffer (100 μg/ml) and denatured at 100°C for 5 min. Ice-cold PBS (8 mM Na_2_HPO_4_, 1.5 mM KH_2_PO_4_ (pH 7.2), 137 mM NaCl, 2.7 mM KCl) containing 0.1 M MgCl_2_ was then added to the DNA solution to a final concentration of 10 μg/ml, and aliquots of the resultant single-stranded DNA solution (100 μl/well) were dispensed into a FluoroNunc Plate (Thermo Fisher Scientific Inc., Waltham, MA, USA). The plate was then sealed and incubated for 3 h at 28°C, after which the mixture was discarded and the plate was dried overnight at 48°C. Photobiotin-labeled DNA was then prepared using a PHOTOPROBE (VECTOR lab., Burlingame, CA, USA) according to the manufacturer’s instructions. About 25 ng of labeled DNA were distributed into each well of a microplate, and hybridization was carried out at for 3 h at 40°C in the hybridization mixture (2×SSC, 5×Denhardt, 3% dextran sulfate, 50% formamide and 200 μg of denatured salmon sperm DNA). Streptavidin-conjugated horseradish peroxidase (Thermo Fisher Scientific Inc.) was diluted 2000-fold in PBS containing 0.5% bovine serum albumin and 0.1% Triton X-100, and 100 μ1 of the diluted enzyme solution were added to each well. The plate was then incubated for 30 min at 37°C and washed three times in 300 μ1 of 2xSSC. After washing, 100 μ1 of chromogenic substrate solution (0.1 M citric acid-0.2 M Na_2_HPO_4_ (pH 6.2)-10% DMFO containing of 2% 3, 3', 5, 5', tetramethylbenzidine in DMFO and 2% H_2_O_2_ aqueous (0.3%)) were added to each well. After incubating the samples and standards for 10 min, the absorbance at 655 nm was read on a Multiskan FC microplate reader (Thermo Fisher Scientific Inc.).

## Results

### Phenotypic characterization of the isolates

**Figure 1:**
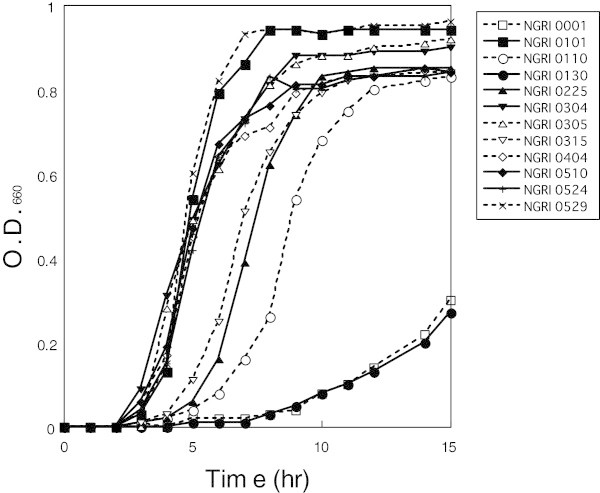
**Growth curves for isolated silage-making LABs.** Cells were grown at 37°C for 15 h in MRS media and monitored based on absorbance at 660 nm. Relative cell densities are an average of four independent cultures.

All of the isolates grew in MRS broth at 30-43°C, and strains NGRI 0001, 0110 and 0130 grew at 45°C (data not shown). When cultivated at 37°C, strains NGRI 0101 and 0529 reached the stationary phase within 8 h after inoculation (Figure 1). With the exception of strains NGRI 0130 and 0305, the isolated strains were homofermentative and did not produce gas from glucose. The peptidoglycans of strains NGRI 0110, 0130, 0304 and 0305 were the non-diamino pimericacid type, while those of the other strains were diamino pimericacid. None of the strains was able to assimilate soluble starch, and the sugar utilization properties of strains NGRI 0001, 0315 and 0524 were identical. Based on their properties, strain NGRI 0110 was coarsely categorized as a *L. casei* group, and strains NGRI 0001, 0101, 0225, 0315, 0404, 0524 and 0529 were categorized as a *L. plantarum* group. Strains NGRI 0304, 0305 and 0510 were grouped together as *Pediococcus* species. That said, the properties of the isolates were not all identical to those of the reference strains.

### Analysis of the V2-V3 variable region of 16S rDNA and the 16S-23S rDNA ITS region

**Figure 2:**
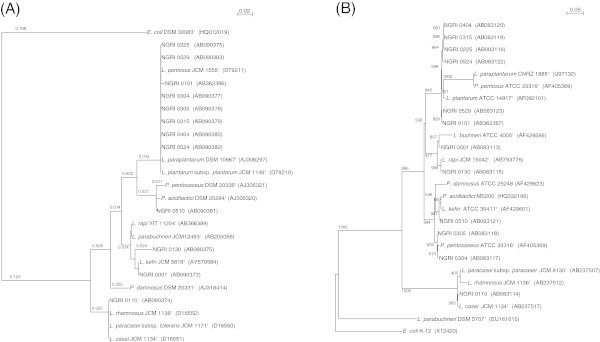
**Phylogenetic trees constructed using the neighbor-joining method with the sequences of the V2-V3 region of the 16S rRNA gene (A) and the ITS region (B) from the isolated LAB strains and their taxonomic neighbors.** The V2-V3 gene sequence from *E. coli* DSM 30083^T^ (HQ012019) was used as the outgroup in **(A)**. The ITS sequence from *E. coli* K-12 (X12420) was used as the outgroup in **(B)**. Bootstrap values based upon 1000 replicates are included at the major branch points. The bars show the number of nucleotide substitutions per site.

An approximately 200-bp band amplified using primers HDA1 and HDA2 was detected in all LAB isolates tested (data not shown). The nucleotide sequences from the isolated strains NGRI 0225, 0304, 0305, 0315, 0404, 0524 and 0529 showed 100% identity to those of *L. plantarum*, *L. paraplantarum* and *L. pentosus*. The highest sequence homology shown by strain NGRI 0110 was 99% to *L. casei*, *L. paracasei* subsp. *tolerans* and *L. rhamnosus*, while strain NGRI 0510 showed 99%, 98% and 92% identity to *P. acidilactici, P. pentosaceus* and *P. damnosus*, respectively. The nucleotide sequence in the V2-V3 region of strain NGRI 0001 perfectly matched that of *L. kefri*. The sequences from strain NGRI 0130 showed significant identity to the V2-V3 regions of *L. rapi* and *L. parabuchneri*. Using these partial 16S rRNA sequences, we constructed a phylogenetic tree (Figure 2(A)). Four clusters were formed at a similarity level of 97%, with the *L. plantarum* cluster containing the largest number of isolates (8) and the *L. casei* cluster containing the fewest (1). Strains NGRI 0001 and 0130 were categorized in the *L. kefri* cluster.

Amplification of the ITS region using primers 16A-1 and 23B-1 yielded an approximately 540-bp band with all samples tested (data not shown). The ITSs of strains NGRI 0225, 0315, 0404 and 0524, showed 99%, 100% and 100% identity to those of *L. plantarum*, *L. pentosus* and *L. paraplantarum*, respectively, while the ITSs of strains NGRI 0304 and 0305 showed 99%, 95% and 92% identity to the those of *P. pentosaceus*, *P. acidilactici* and *P. damnosus*, respectively. Using the ITS sequences, we constructed a phylogenic tree, which included four clusters: an *L. plantarum* group, *L. casei* group, *L. buchneri* group and *Pediococcus* group (Figure 2(B)).

The dendrograms for both the V2-V3 and ITS regions did not separate the positions of strains closely related on the evolutionary tree, such as among *L. plantarum*, *L. paraplantarum* and *L. pentosus*, or among *L. casei*, *L. paracasei* and *L. rhanmosus*. The sequences of the16S rRNA V2-V3 regions of strains NGRI 0101, 0315, 0404, 0524 and 0529 were identical to those of *L. paraplantarum*, *L. pentosus* and *L. plantarum*. However, the isolated strains were able to utilize raffinose, which is not fermented by *L. paraplantarum* (Curk et al. 1996) (Table 2). Therefore, these strains should be *L. pentosus* or *L. plantarum.* The V2-V3 and ITS sequences of the isolates have been deposited in the DNA Data Bank of Japan (DDBJ) under accession numbers AB090373 to AB090383 and AB083113 to AB083123, respectively.

**Table 2:** **Characteristics of isolated strains**

Strain	NGRI 0001	NGRI 0101	NGRI 0110	NGRI 0130	NGRI 0225	NGRI 0304	NGRI 0305	NGRI 0315	NGRI 0404	NGRI 0510*	NGRI 0524	NGRI 0529
Shape	Rods	Rods	Rods	Rods	Rods	Cocci	Cocci	Rods	Rods	Cocci	Rods	Rods
Gas from glucose	-	-	-	+	-	-	+	-	-	-	-	-
Growth at 15°C	+	+	+	+	+	+	+	+	+	+	+	+
Growth at 45°C	+	-	+	+	-	-	-	-	-	-	-	-
Fermentation type	Homo	Homo	Homo	Hetero	Homo	Homo	Hetero	Homo	Homo	Homo	Homo	Homo
Lactate formed	DL	DL	L	DL	DL	L	DL	DL	DL	DL	DL	DL
Peptidoglycan type	A_2_pm	A_2_pm	Non- A_2_pm	Non- A_2_pm	A_2_pm	Non- A_2_pm	Non- A_2_pm	A_2_pm	A_2_pm	A_2_pm	A_2_pm	A_2_pm
Acid from:												
Na-Gluconate	+	+	+	+	+	+	+	+	+	+	+	+
L- Arabinose	+	-	+	+	-	+	+	+	+	+	+	+
Lactose	+	+	+	-	+	-	-	+	+	-	+	+
Galactose	+	+	+	-	+	+	+	+	+	+	+	+
Sucrose	+	+	+	-	+	+	+	+	+	-	+	+
Mannose	+	+	+	-	+	+	+	+	+	+	+	+
Fructose	+	+	+	-	+	+	+	+	+	+	+	+
Soluble starch	-	-	-	-	-	-	-	-	-	-	-	-
D-Xylose	+	-	-	+	-	-	+	+	+	+	+	+
D-Ribose	+	+	+	+	+	+	+	+	+	+	+	+
Mannitol	+	+	+	-	+	-	+	+	+	+	+	+
Sorbitol	+	+	+	-	+	+	-	+	+	+	+	+
Rhamnose	+	-	+	-	-	+	-	+	-	+	+	-
Trehalose	+	+	+	-	+	+	+	+	+	-	+	+
Raffinose	+	+	-	+	+	+	+	+	+	-	+	+
DNA G+C content (%)	45.1	45.3	47.3	46.2	45.1	39.7	39.0	44.4	45.1	41.0	46.1	44.0

*Data was obtained from Doi et al (2009).

### Genotyping using RAPD-PCR profiles

**Figure 3:**
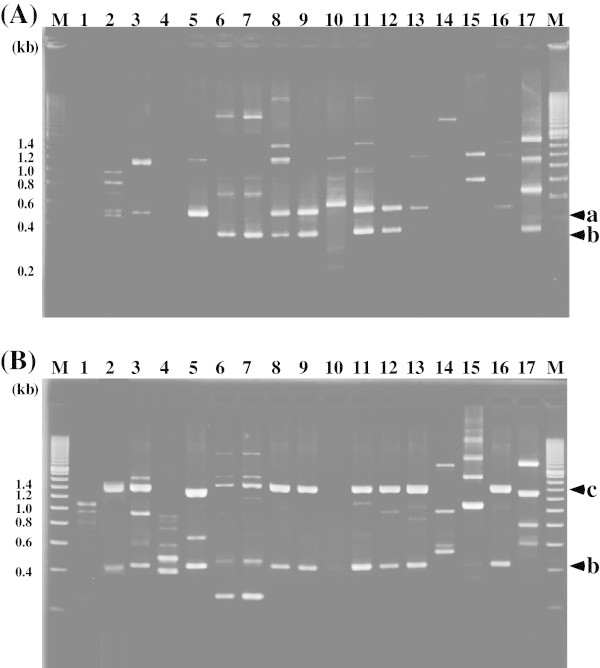
**RAPD-PCR fingerprinting of isolated strains and related species using primers AP-A-2 (A) and AP-A-23 (B).** Lanes: M, size markers (200 bp DNA ladder; TOYOBO); 1, NGRI 0001; 2, NGRI 0101; 3, NGRI 0110; 4, NGRI 0130; 5, NGRI 0225; 6, NGRI 0304; 7, NGRI 0305; 8, NGRI 0315; 9, NGRI 0404; 10, NGRI 0510; 11, NGRI 0524; 12, NGRI 0529; 13, *L. plantarum* JCM 1057; 14, *L. casei* JCM 1134^T^; 15, *L. rhamnosus* JCM 1136^T^; 16, *L. plantarum* JCM 1149^T^; 17, *L. pentosus* JCM 1558^T^.

RAPD-PCR analyses were performed using single oligonucleotide primers (10 mer). No amplified fragment was generated with the AP-A-6 or AP-A-8 primer (data not shown). On the other hand, the genotypes obtained with the AP-A-2 (Figure 3(A)), AP-A-19 (data not shown) and AP-A-23 (Figure 3(B)) primers were completely consistent with the phenotypes of the isolates. The amplified patterns were detectable in all isolates and reference strains, and RAPD-PCR profiles recognized some clusters. In Figure 3(A), a 0.5-kb fragment (arrowhead a) was amplified in the *L. plantarum* strains JCM 1057 and JCM 1149^T^ (lanes 13 and 16) using primer AP-A-2, and equal-sized bands were observed in lanes 2, 3, 5, 8, 9, 11, 12 and 13, suggesting the amplified fragments are common to all *L. plantarum* strains. Although amplification of a 0.3-kb fragment (arrowhead b) was also observed in lanes 6, 7, 8, 9, 11, 12 and 17, no other amplified fragments were identical to *L. pentosus* JCM 1558^T^ (lane 17). Moreover, the profiles of strains NGRI 0304 and 0305 (lanes 6 and 7) were virtually identical to one another, but did not resemble the profiles of any other strains. In Figure 3(B), two major bands, at 1.4 kb (arrowhead c) and 0.4 kb (arrowhead d), appeared in the *L. plantarum* JCM 1057 (lane 13) and *L. plantarum* JCM 1149^T^ (lane 16) profiles obtained using primer AP-A-23. Similar patterns were observed with strains NGRI 0101, 0225, 0315, 0404, 0524 and 0529 (lanes 2, 5, 8, 9, 11 and 12), suggesting these major amplified bands are indicative of the *L. plantarum* group.

### Immuno-identification using anti-lactobacilli antibodies

**Figure 4:**
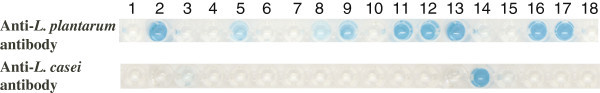
**Immuno-identification of** ***L. plantarum*** **and** ***L. casei*** **group strains.** Lanes: 1, NGRI 0001; 2, NGRI 0101; 3, NGRI 0110; 4, NGRI 0130; 5, NGRI 0225; 6, NGRI 0304; 7, NGRI 0305; 8, NGRI 0315; 9, NGRI 0404; 10, NGRI 0510; 11, NGRI 0524; 12, NGRI 0529; 13, *L. plantarum* JCM 1057; 14, *L. casei* JCM 1134^T^; 15, *L. rhamnosus* JCM 1136^T^; 16, *L. plantarum* JCM 1149^T^; 17, *L. pentosus* JCM 1558^T^; 18, *E. coli* DH5α.

The antigenicity of the LAB isolates to anti-*L. plantarum* and anti*-L. casei* antibodies was assessed using an enzyme-linked immunosorbent assay (ELISA). Six samples from silage (NGRI 0101, 0225, 0315, 0404, 0524 and 0529) and three reference samples (*L. plantarum* strains JCM 1057 and JCM 1149^T^, and *L. pentosus* JCM 1558^T^) reacted positively with the anti-*L. plantarum* antibody (Figure 4(A)). In addition, one reference sample (*L. casei* JCM 1134^T^) reacted with the anti-*L. casei* antibody. *L. rhamnosus* JCM 1136^T^, which is a *L. casei*-related strain, showed a negative response to the antibody (Figure 4(B)). Among the LAB isolates tested, only NGRI 0110 cells showed faint positivity for the anti-*L. casei* antibody. These results suggest that the anti-*L. casei* antibody tested could differentiate between *L. casei* and *L. rhamnosus*, and that there was no strain which could be identified as *L. casei* among the 12 LAB isolates.

### Whole-cell protein fingerprinting

**Figure 5:**
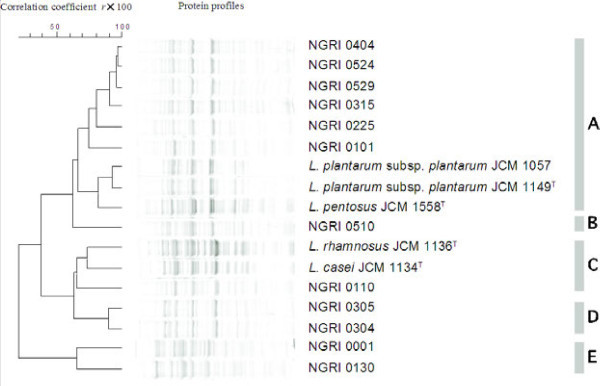
**Protein profiles of representative LAB isolates and reference strains.** The left-hand side of the figure shows the mean correlation coefficients expressed as percentages (*r*×100) and represented as a dendrogram calculated through UPGMA analysis of the selected strains. The right-hand side of the figure shows the delineation of the 5 clusters.

Whole-cell protein fingerprinting of the 12 isolates and 5 reference strains yielded distinctly different band patterns (Figure 5), with an intragel reproducibility of approximately 90%. Numerical analysis of the SDS-PAGE patterns obtained with the isolated strains resolved 5 clusters (A-E), emerging at a similarity level of 60%, and confirmed the phenotypic characterization of the isolates. Cluster A includes *L. plantarum* and *L. pentosus*, while cluster C includes *L. casei* and *L. rhamnosus*.

### Analysis of G+C content and DNA-DNA relatedness

The G+C content of the isolates ranged from 39.0% to 47.3% (Table 2). Particularly noteworthy was the finding that the G+C contents of NGRI 0101, 0315, 0404, 0524 and 0529 were all around 45 mol%, which is consistent with those of *L. plantarum*, whereas strains NGRI 0304 and 0305 showed lower G+C contents (less than 40%).

DNA relatedness was analyzed using the isolated strains along with the 6 type strains, *L. casei* JCM 1134^T^, *L. plantarum* JCM 1149^T^, *L. rhamnosus* JCM 1136^T^, *L. pentosus* JCM 1558^T^, *P. acidilactici* JCM 8797^T^ and *P. pentosaceus* JCM 5890^T^ (Table 3). Levels of DNA relatedness among strains NGRI 0001, 0130 and 0510 and the 5 type strains were lower than 57%. This clearly indicates that these isolates do not belong to the *L. casei* group, *L. plantarum* group or *Pediococcus* species, as these values are well below the 70% threshold for definition of bacterial species (Wayne *et al.*^1987^). Strains NGRI 0101, 0315, 0404, 0524 and 0529 all showed high levels of DNA relatedness (> 70%) with *L. plantarum* JCM 1149^T^, while strains NGRI 0110 and 0225 showed more than 80% DNA relatedness to *L. rhamnosus* JCM 1136^T^ and *L. pentosus* JCM 1558^T^.

**Table 3:** **Levels of DNA-DNA homology for isolated strains**

	DNA-DNA reassociation % with																	
Strain	NGRI 0001	NGRI 0101	NGRI 0110	NGRI 0130	NGRI 0225	NGRI 0304	NGRI 0305	NGRI 0315	NGRI 0404	NGRI 0510	NGRI 0524	NGRI 0529	JCM 1134^T^	JCM 1136^T^	JCM 1149^T^	JCM 1558^T^	JCM 5885^T^	JCM 5890^T^
*L. casei* JCM 1134^T^	30.2	34.1	45.3	40.1	20.3	21.9	19.8	25.1	18.2	10.9	25.2	36.2	100	-	-	-	-	-
*L. rhamnosus* JCM 1136^T^	28.6	26.4	**89.3**	36.2	21.3	14.2	26.7	12.4	21.5	56.3	42.3	31.2	-	100	-	-	-	-
*L. plantarum* JCM 1149^T^	32.3	**83.9**	10.2	15.3	25.6	18.9	21.5	**90.1**	**82.3**	12.9	**79.2**	**84.3**	-	-	100	-	-	-
*L. pentosus* JCM 1558^T^	28.3	30.2	11.2	19.3	**82.9**	34.2	45.6	42.3	35.2	26.3	39.1	36.8	-	-	-	100	-	-
*P. pentosaceus* JCM 5890^T^	10.8	12.8	17.6	20.3	21.6	**83.1**	**87.1**	18.1	16.2	17.3	15.9	14.3	-	-	-	-	100	-
*P. acidilactici* JCM 8797^T^	15.6	14.9	17.1	20.5	12.3	16.5	23.1	13.7	18.7	19.3	18.1	23.8	-	-	-	-	-	100

- Not tested.

Identities over 70% were indicated by boldface.

The experimental results summarized above confirmed *L. plantarum* NGRI 0101, *L. rhamnosus* NGRI 0110, *L. pentosus* NGRI 0225, *P. pentosaceus* NGRI 0304, *P. pentosaceus* NGRI 0305, *L. plantarum* NGRI 0315, *L. plantarum* NGRI 0404, *L. plantarum* NGRI 0524 and *L. plantarum* NGRI 0529. However, because strains NGRI 0001, NGRI 0130 and NGRI 0510 showed significant similarities to the reference strains used, it seemed best to analyze the complete nucleotide sequences of their 16S rRNA gene.

### Phylogenetic analysis of the complete 16S rRNA gene sequence

**Figure 6:**
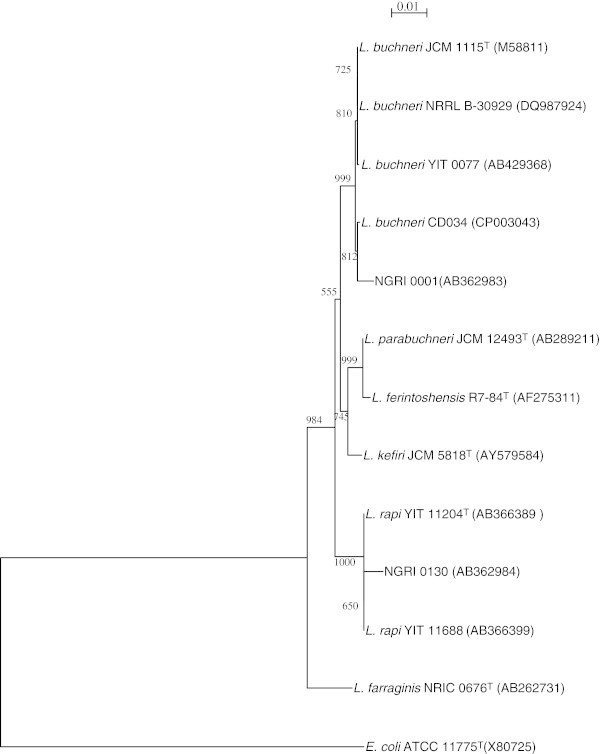
**Phylogenetic trees constructed using the neighbor-joining method with the full-length16S rRNA gene sequences from the isolated LAB strains and their taxonomic neighbors.** The gene sequence of 16S rRNA from *E. coli* ATCC 11775 (X80725) was used as the outgroup. Bootstrap values based upon 1000 replicates are included at the major branch points. The bars show the number of nucleotide substitutions per site.

The complete nucleotide sequences of the 16S rRNA coding genes were determined for two isolated strains, NGRI 0001 and NGRI 0130, which were not identified in the experiments summarized above. The full 16S rDNA sequences of the isolates have been deposited in the DDBJ under accession numbers AB362983 and AB362987. Blast analysis revealed that the 16S rRNA gene sequence of strain NGRI 0001 showed similarity with those of *L. buchneri* CD034 (CP003043) (98.64%), *L. buchneri* NRRL B-30929 (CP002652) (98.57%) and *L. buchneri* JCM 1115^T^ (NR_041293) (98.54%), while the sequence of strain NGRI 0130 showed similarity to those of *L. rapi* YIT 11204^T^ (NR_041659) (98.9%), *L. rapi* YIT 11688 (AB366399) (98.9%) and *L. buchneri* CD034 (97.5%) (Figure 6). From these results, strains NGRI 0001 and NGRI 0130 were identified as *L. buchneri* and *L. rapi*, respectively.

## Discussion

In tropical and subtropical regions, the ensiling process often yields only poor quality silage. Two of the reasons are butyric fermentation by butyric acid bacteria and consumption of the nutrients in the silage by coliform bacteria (Tanaka et al. 1994). Butyric acid is usually produced at high temperatures (30-50°C) by butyric acid bacteria, which have the ability to grow under those conditions. Therefore, to prevent proliferation of butyric acid bacteria and coliform bacteria, silage-making LAB must be capable of rapid growth and high production of lactic acid at high temperatures. Much effort has gone into improving the quality of silage (Weinberg et al. 2007; Lindsey and Kung Jr. 2010; Parvin et al. 2010). In those studies, LABs grown at room temperature showed great ability to improve the quality of silage. However, it was unclear whether strains showing high lactic acid production could grow ahead of butyric acid bacteria or coliform bacteria at higher temperatures (>37°C), which will be necessary for potentially useful candidates to adapt to tropical and subtropical regions. To prepare potential inoculants for ensilage at high temperature, we isolated LABs from silage prepared in southwestern Japan, where the weather can be hot and humid.

The 12 strains of silage-making LABs tested showed distinct phenotypes that were irreconcilable with the reference strains, which included the type strains of *L. plantarum*, *L. pentosus*, *L. casei* and *L. rhamonsus*, 5 strains frequently used in silage preparation and frequently isolated from silage (Filya et al. 2000; Nishino et al. 2004; Okine et al. 2005; Klocke et al. 2006). It was difficult to distinguish between *L. plantarum* and its related species, *L. pentosus*, and between *L. casei* and *L. rhamonsus.* Although we applied phage typing to these closely related LAB strains (Doi et al. 2003), we were unable to do a complete identification. We therefore attempted to identify the isolated LABs by comparing the sequences of the 16S-23S rRNA ITS and the 16S rRNA V2-V3 region. Both approaches enabled identificat-ion of several clusters, including the *L. plantarum* and *L. casei* groups, among others (Figure 2). Although strain NGRI 0110 was distributed to the *L. casei* group, it was impossible to distinguish among *L. casei*, *L. paracasei* and *L. rhamonsus.* Strains NGRI 0304 and 0305, which were grouped with *P. pentosaceus* based on their ITS sequences, were categorized to the *L. plantarum* group based on their V2-V3 region sequences. These results indicate that although we were able to classify the isolated LABs at the genus level, we could not resolve them at the species level.

RAPD-PCR profiles obtained using primers AP-A-2 and AP-A-23 enabled us to distinguish among closely related silage-making LABs when *L. casei*, *L. rhamonsus, L. plantarum* and *L. pentosus* were used as references. The RAPD-PCR patterns of *L. plantarum* (lanes 13 and 15 in Figure 3) obtained with primer AP-A-23 were identical, and the patterns in lanes 2, 8, 9, 11 and 12 were identified as *L. plantarum*. These primers thus show a potential for *L. plantarum*-specific typing, along with those reported previously (Johansson et al. 1995; Quere et al. 1997). With the 25 primers used in this study, however, we were unable to resolve profiles specific for *L. rhamnosus* or *L. pentosus*.

There have been few studies involving the immuno-identification of bacteria using monoclonal antibodies, and those that have been done focused mainly on pathogenic bacteria (Hearty et al. 2006; Klesius et al. 2006). With respect to LABs, only immuno-identification of *L. casei* strain Shirota in the gastrointestinal tract has been reported (Yuki et al. 1999). In the present study, an anti-*L. plantarum* monoclonal antibody reacted positively with species belonging to *L. plantarum* (NGRI 0101, 0315, 0404, 0524, 0529 and JCM 1149^T^) and *L. pentosus* (NGRI 0225 and JCM 1558^T^) (Figure 4(A)). According to the manufacturer, this antibody reacts positively with only *L. plantarum* among 25 strains of LAB, 2 strains of yeast and 5 other microorganisms; however, its reactivity with *L. pentosus* has never been tested. From the above results, we suggest this antibody could be used to identify *L. plantarum* and *L. pentosus*, and that it is impossible to differentiate between those two closely related species. The cell surfaces of *L. plantarum* and *L. pentosus*, which both contain *meso*-diaminopimelic acid in their peptidoglycan, likely have similar structures and antigens. On the other hand, the anti-*L. casei* monoclonal antibody tested could distinguish between *L. casei* JCM 1134^T^ and *L. rhamnosus* JCM 1136^T^. Strain NGRI 0110, which should be identified as *L. rhamnosus*, reacted slightly to this antibody. Differences in the immuno-reactions among the isolates and reference strains were also observed in clustering constructed based on the cell protein profiles (Figure 5). Such variation among *L. casei* and *L. rhamnosus* strains could be caused by cell surface reactions similar to those mediating adhesion to intestinal mucus membranes (Ouwehand et al. 1999).

Dendrograms obtained using SDS-PAGE with whole-cell protein extracts from LAB isolates and reference strains have been applied for rapid differentiation of species from fermented sausages, bread, yoghurt, cheese, plants and human faecal samples (Dimitrov et al. 2005; Ricciardi et al. 2005; Benito et al. 2008). When we applied this method to differentiation of silage-making LAB, we found good agreement between the SDS-PAGE profiles and the physiological and genetic characteristics of the LAB tested. There appeared to be obvious differentiation among the *L. plantarum* group (cluster A), *L. casei* group (cluster C) and *P. pentosaceus* group (cluster D), and strains NGRI 0001, 0130 and 0510 were separated into the known groups. Moreover, since strain NGRI 0510 was identified as *P lolii*, a novel *Pediococcus* species (Doi et al. 2009; Doi et al. 2013), its phylogenetic position may be specific.

From the results of phylogenetic analysis using the complete 16S rRNA sequences, strains NGRI 0001 and 0130 were identified as *L. buchneri* and *L. rapi*, respectively (Figure 6). Because *L. buchneri*, *L. kefiri*, *L. parabuchneri* and *L. rapi* are phylogenetically the most closely related species (Watanabe et al. 2009), this result is consistent with the finding that the partial sequence of the 16S rRNA from strain NGRI 0001 was similar with that from *L. buchneri* and *L. kefiri* (Figure 2A).

In conclusion, our results show that representative strains isolated from silage produced in hot and humid weather contain six strains belonging to *L. plantarum* (NGRI 0101, 0315, 0404, 0524 and 0529), *L. pentosus* (NGRI 0225), *L. rhamonsus* (NGRI 0110), *L. buchneri* (NGRI 0001), *L. rapi* (NGRI 0130) *P. pentosaceus* (NGRI 0304 and 0305) and *P. lolii* (NGRI 0510). Although strain NGRI 0315 was identified as *L. plantarum*, its growth rate was slower than those of other *L. plantarum* strains (Figure 1). Such slow growing strains exhibit greater acid tolerance than rapidly growing strains (data not shown). It therefore seems likely that rapidly growing LABs would produce lactic acid to reduce pH in silage, after which large numbers of cells from acid-tolerant strains living at low pH could competitively inhibit the growth of spoilage microbes, thereby contributing to silage stability. We will report on the application and evaluation of these strains as silage starters adapted to hot and humid weather.
